# *Candida albicans* Modulates Host Defense by Biosynthesizing the Pro-Resolving Mediator Resolvin E1

**DOI:** 10.1371/journal.pone.0001316

**Published:** 2007-12-19

**Authors:** Eric J. Haas-Stapleton, Yan Lu, Song Hong, Makoto Arita, Silvio Favoreto, Santosh Nigam, Charles N. Serhan, Nina Agabian

**Affiliations:** 1 Department of Cell and Tissue Biology, University of California at San Francisco, San Francisco, California, United States of America; 2 Center for Experimental Therapeutics and Reperfusion Injury, Department of Anesthesiology, Perioperative and Pain Medicine, Brigham and Women's Hospital, Harvard School of Dental Medicine and Harvard Medical School, Boston, Massachusetts, United States of America; 3 Department of Oral Medicine, Infection and Immunity, Harvard School of Dental Medicine and Harvard Medical School, Boston, Massachusetts, United States of America; 4 Eicosanoid and Lipid Research Division, Centre for Experimental Gynecology and Breast Research, Charité-University Medical Centre Benjamin Franklin, Berlin, Germany; University of Birmingham, United Kingdom

## Abstract

*Candida albicans* is an opportunistic fungal pathogen of humans that resides commensally on epithelial surfaces, but can cause inflammation when pathogenic. Resolvins are a class of anti-inflammatory lipids derived from omega-3 polyunsaturated fatty acids (PUFA) that attenuate neutrophil migration during the resolution phase of inflammation. In this report we demonstrate that *C. albicans* biosynthesizes resolvins that are chemically identical to those produced by human cells. In contrast to the trans-cellular biosynthesis of human Resolvin E1 (RvE1), RvE1 biosynthesis in *C. albicans* occurs in the absence of other cellular partners. *C. albicans* biosynthesis of RvE1 is sensitive to lipoxygenase and cytochrome P450 monoxygenase inhibitors. We show that 10nM RvE1 reduces neutrophil chemotaxis in response to IL-8; 1nM RvE1 enhanced phagocytosis of *Candida* by human neutrophils, as well as intracellular ROS generation and killing, while having no direct affect on neutrophil motility. In a mouse model of systemic candidiasis, RvE1 stimulated clearance of the fungus from circulating blood. These results reveal an inter-species chemical signaling system that modulates host immune functions and may play a role in balancing host carriage of commensal and pathogenic *C. albicans*.

## Introduction

Oxygenated derivatives of poly unsaturated fatty acids (PUFAs) play important roles in the regulation of development, wound healing and defensive responses among diverse taxa, including plants and humans. In humans, resolvins, lipoxins and prostaglandins promote or resolve inflammation [1], [2], [3] while in plants, oxylipins mediate a wider range of physiological activities [4], [5], [6]. The resolution of inflammation is an essential process that activates specific cellular pathways to prevent chronic inflammation and inappropriate tissue damage. In humans, oxygenated mediators derived from omega-3 PUFAs are among the first potent counter-regulatory signaling molecules identified to promote the resolution of inflammation [7], [8]. The observation that the genome of the opportunistic fungal pathogen, *Candida albicans*, encodes a wider range of fatty acid-utilizing enzymes as compared with the non-pathogenic Brewer's yeast, *Saccharomyces cerevisiae* [9], prompted us to evaluate the range of oxygenated lipids produced by *C. albicans* cultured in the presence of the omega-3 PUFAs eicosapentaneioc acid [EPA] and docosahexaenoic acid [DHA]. In this report we show that *C. albicans* is capable of *de novo* biosynthesis of Resolvin E1 (RvE1), a potent anti-inflammatory mediator [10], from EPA. Previously, RvE1 was described only in inflammatory exudates of mouse and human. In humans, RvE1 selectively interacts with both the leukotriene B4 receptor (BLT1) and the G-protein coupled receptor ChemR23 expressed on the surface of neutrophils [11] to promote the resolution of dermal inflammation, peritonitis and colitis in murine models of these diseases [10], [12], [13]. Biosynthesis of RvE1 in humans is a *trans*-cellular process involving endothelial cell cytochrome P450 monooxygenase enzymes (CYP450) that catalyze conversion of EPA to 18-HEPE, which is further oxygenated by neutrophil 5-lipoxygenase and other enzymes to generate RvE1 [14]. RvE1 is also formed during multi-species interactions, such as during inflammation resulting from microbial infections. In that instance, microbial CYP450s capable of converting EPA into 18-HEPE [15] would provide the substrate used by neutrophils for RvE1 synthesis.

*Candida albicans* is a dimorphic fungus and is the leading cause of invasive fungal infections among hospitalized patients in the United States [16]. However, in the majority of healthy individuals, *C. albicans* is a commensal organism, persisting as a benign saprophyte on mucosal epithelial surfaces [17]. In immunocompromised or therapeutically immunosuppressed patients (ex. HIV infection or cancer treatment), this opportunistic pathogen can become invasive, penetrating the upper layers of the mucosa and causing localized inflammation [18]. If provided access to the blood stream, as with the use of catheters or other prosthetic devices, the fungus can disseminate to a wide range of organs, resulting in often-fatal hematogenous disease [19]. In both healthy and immunocompromised individuals, the innate immune system, and neutrophils in particular, provide the first line of host defense against *Candida* infections, which is consistent with the high incidence of candidiasis in neutropenic patients [20], [21]. Unlike other cells in the host innate armamentarium, neutrophils ingest and kill both yeast and hyphal phenotypes of *Candida* [22]. The primary effector functions of neutrophils important for limiting invasion by *C. albicans* and resolving inflammation include phagocytosis, the generation of reactive oxygen species (ROS) and fungal killing [23]. Epithelial and endothelial cells participate in innate defense as well by secreting cytokines, including interleukin-8 (IL-8), which serve as a chemotactic signal, attracting neutrophils to sites of inflammation [24], [25], [26], [27].

In this study, we show that *C. albicans* biosynthesizes RvE1 *de novo* from EPA, the RvE1 being indistinguishable from RvE1 produced by its human host. In the context of infection, RvE1 attenuates IL8-mediated neutrophil migration while stimulating neutrophil phagocytosis, intracellular ROS generation, and killing of *Candida* both *in vitro* and circulating in the blood. These findings suggest that RvE1 stimulates clearance and resolution of pathogenic *Candida* infections as well as evoking local anti-inflammatory responses. In this manner, a chemical signaling mechanism, based on a bioactive lipid mediator shared by both host and pathogen, provides a novel interspecies communication system that, in the case of *Candida*, may modulate its commensal to virulent transition in a vulnerable host.

## Results

### *C. albicans* biosyntheses of oxygenated derivatives of EPA and DHA

**Figure 1:**
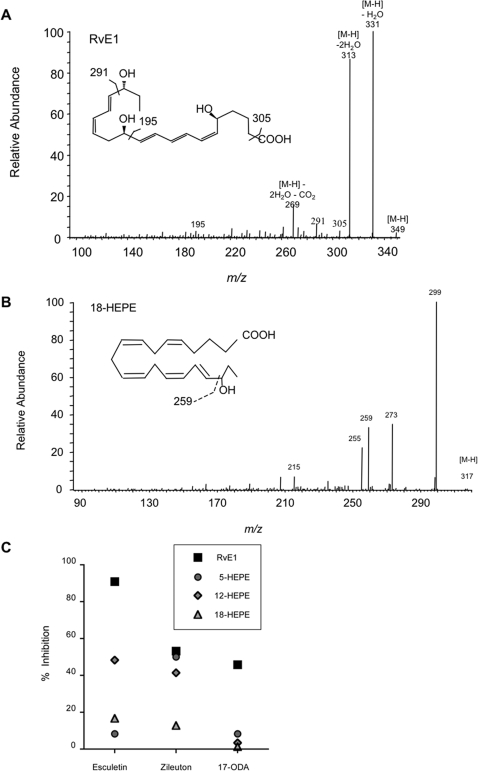
* C. albicans* produces a tri-hydroxy derivative of EPA that is structurally identical to the human anti-inflammatory lipid mediator RvE1. The MS/MS spectra of biogenic RvE1 (1A) and 18-HEPE (1B) produced by *C. albicans* cultured in liquid media supplemented with EPA. (1C) *C. albicans*, cultured in the presence of EPA the LO inhibitors esculetin or zileuton (100 µM) as well as the CYP450 inhibitor, 17-ODA, reduced RvE1 biosynthesis (squares). LO inhibitors, but not the CYP450 inhibitor, reduced biosynthesis of 5-HEPE (circles), 12-HEPE (diamonds), and 18-HEPE (triangles).

The genome of *C. albicans* encodes a large number of oxidative and lipid-utililzing enzymes as compared with that of the non-pathogenic yeast *Saccharomyces cerevisiae,* [9] which suggested to us that lipids and lipid oxidation may play an important role in *C. albicans* pathogenesis and cell biology. As might be predicted from these genomic characteristics, we were able to propagate *C. albicans* in media supplemented with fatty acids comprised of eighteen to twenty-two carbon chains as sole carbon source (not shown). When provided with complex oils such as olive, fish and flaxseed oil as their sole carbon, *C. albicans* exhibited robust growth, rivaling that obtained in standard glucose-containing media (Figure S1). To understand how these lipids were being utilized by *Candida,* we assayed the range of oxygenated lipid metabolites produced by *C. albicans.* After culture in the presence of the essential omega-3 PUFA EPA or DHA, we detected a large and varied repertoire of oxygenated lipids as assayed by liquid chromatography-tandem mass spectrometry (LC-MS/MS) (Table S1). Notable among the metabolites produced by *C. albicans* was the potent anti-inflammatory lipid mediator RvE1 and its biosynthetic precursors, 18-hydroxyeicosapentaenoic acid (HEPE), 15-HEPE and 5-HEPE (Figure 1AB and Table S1). For biogenic RvE1 produced by *C. albicans*, the base peak [M-H] ion *m/z* 349, and fragment ions *m/z* 195, 291, and 305, were identical to those derived from synthetic RvE1 and biogenic RvE1 isolated from human plasma [10]. These results demonstrate that the RvE1 produced by *C. albicans* is chemically identical to that produced by humans. Additionally, the base peak [M-H] ion *m/z* 317, with fragment ion *m/z* 259, was identical to that of synthetic 18-HEPE (Figure 1B), suggesting that *C. albicans* lipid metabolites may also provide precursors for mammalian cell synthesis of RvE1 and other lipid mediators.

To explore the potential enzymatic pathways associated with RvE1 biosynthesis, *C. albicans* was cultured in the presence of EPA with and without a panel of well-appreciated inhibitors of mammalian lipoxygenase (LO) [esculetin (12/15-LO inhibitor) and zileuton (5-LO inhibitor)] as well as a cytochrome P450 monooxygenase (CYP450) inhibitor [17-octadecynoic acid (17-ODA)]. The effect of these inhibitors upon the biosynthesis of RvE1 and its biosynthetically-related products was analyzed using LC/MS-MS. *C. albicans* cultured in the presence of EPA with vehicle (<0.1% ethanol) served as control. Culture in the presence of EPA and either esculetin or zileuton (100 µM) resulted in a 91% or 53% reduction in the level of RvE1 synthesized in *C. albicans*, respectively. In the presence of 100 µM 17-ODA, we observed a 46% reduction in RvE1 biosynthesized and minimal reductions in the biosynthesis of RvE1 precursors (1–8% inhibition). No significant amount of any oxygenated lipids was detected in the supernatant or cell pellet of *C. albicans* cultured with dextrose as the sole carbon source (data not shown). These specific inhibitors of mammalian LO and CYP450 had no observed effect on fungal growth or morphology (Figure S2).

Using an *in silico* approach to identify the fungal genes encoding this putative LO activity, we performed detailed TBLASTN analysis (September 2007) using known 3-LO, 4-LO, 5-LO, 8-LO, 12-LO, 15-LO, and ALOX protein sequences from a wide range of organisms including human, mouse, rice, soybean, potato, and bacteria. These comparisons failed to identify any open reading frames in the currently available *C. albicans* genome databases with significant homology to LO sequences.

### RvE1 blocks IL-8-stimulated neutrophil chemotaxis

**Figure 2:**
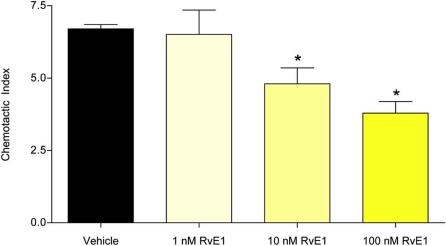
RvE1 blocks IL-8-stimulated neutrophil chemotaxis. IL-8-directed chemotaxis of neutrophils was significantly inhibited by 10 and 100 nM RvE1 (Asterisks indicate significant differences from vehicle-treated controls; ANOVA: p<0.001).

To study the effect of RvE1 on neutrophil chemotaxis, we first assayed the chemotactic response of neutrophils in response to IL-8. The chemokine was placed in the lower chamber of a Boyden apparatus (Transwell), and neutrophils were placed in the upper chamber. As expected, IL-8 functioned as an attractant, resulting in the migration of neutrophils into the lower chamber (Figure 2; chemotactic index = 6.7 +/− 0.26). However, incubation of neutrophils with increasing concentrations of RvE1 (1.0–100 nM) resulted in a concentration-dependent inhibition of IL-8-stimulated neutrophil migration into the lower chamber of the Transwells. Both 10 nM and 100 nM RvE1 produced significant reductions in IL-8-directed migration when compared to controls (ANOVA: For 10 nM and 100 nM, p<0.001; Figure 2). Results from two additional experiments using different blood donors produced similar results (Figure S3). RvE1 alone (1–100 nM) did not stimulate neutrophil motility—neither chemotaxis, fugetaxis, nor chemokinesis (Figure S4).

### RvE1 enhances effector functions of neutrophils

**Figure 3:**
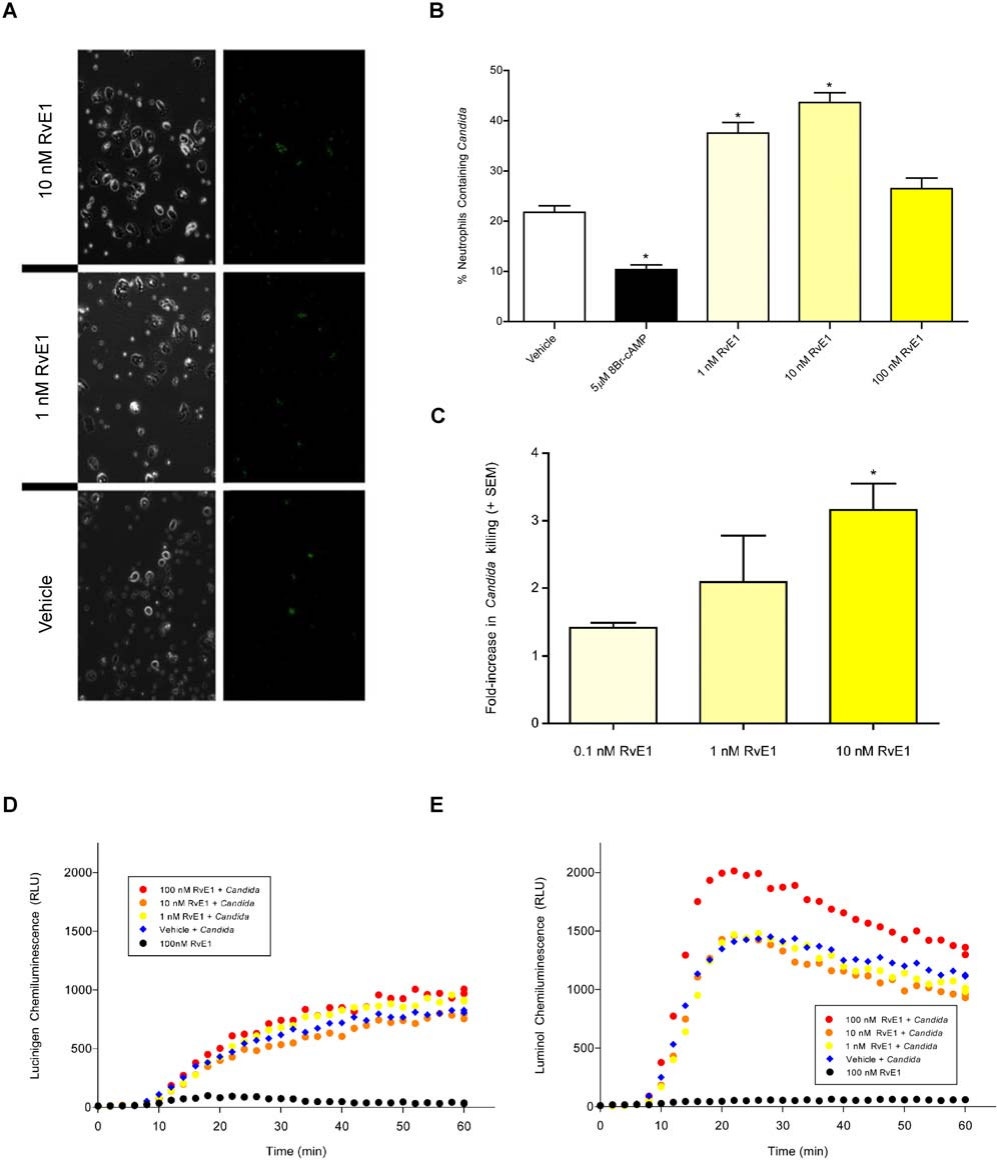
RvE1 enhances the effector functions of neutrophils. (3AB) Neutrophil phagocytosis of FITC-stained heat-killed opsonized (HKO) *C. albicans.* (3B) Significantly more FITC-stained HKO *C. albicans* were phagocytosed by adherent human neutrophils treated with RvE1 (yellow bars) relative to vehicle-treated cells (open bar; asterisks indicate significant differences from vehicle-treated controls; ANOVA: p<0.001). Neutrophils treated with the non-hydrolyzable cAMP analog 8-bromo-cAMP were less likely to phagocytose the yeast (black bar). (3A) Representative images of isolated neutrophils phagocytosing FITC-stained HKO *C. albicans* in the presence of 10 nM RvE1, 1 nM RvE1 or vehicle and trypan blue. Green fluorescence (right panels) correspond to FITC-stained HKO *C. albicans* phagocytosed by neutrophils (3C) Neutrophils exposed to RvE1 exhibited a positive dose-dependent increase in their ability to kill opsonized *C. albicans* (yellow bars). Twice as many *C. albicans* were killed by neutrophils treated with 1nM RvE1 and over three times as many yeast were killed by neutrophils treated with 10 nM RvE1 relative to vehicle-treated neutrophils. Asterisks indicate significant differences from vehicle-treated controls (ANOVA: p<0.001). (3D) RvE1 had no effect on hydroxy-radical produced by neutrophils exposed to HKO *C. albicans* (red, orange, and yellow circles vs blue diamonds). (3E) 100 nM RvE1 increased neutrophil super-oxide production in neutrophils exposed to HKO *C. albicans* relative to neutrophils exposed to vehicle and HKO *C. albicans* (red circles vs blue diamonds) while lower concentrations of RvE1 (orange and yellow circles) did not increase neutrophil super-oxide relative to cells exposed to vehicle and HKO *C. albicans* (blue diamonds). 100nM RvE1 alone did not increase the amount of ROS produced by neutrophils relative to vehicle controls (3DE: black circles).

The primary effector functions of neutrophils important for limiting invasion by *C. albicans* and resolving inflammation include phagocytosis, ROS generation and fungal killing [23]. In light of the inhibition of IL-8-dependent neutrophil chemotaxis by RvE1, the effect of RvE1 on each of these other properties of neutrophils were measured. RvE1 enhanced each of these functions in neutrophils exposed to *C. albicans* (Figure 3). Phagocytosis of *C. albicans* was assayed using FITC-stained heat-killed opsonized (HKO) yeast cells. As shown in Figure 3AB, significantly more *Candida* were phagocytosed by adherent human neutrophils treated with RvE1 relative to vehicle-treated cells [Figure 3B; ANOVA: For 1 and 10nM RvE1, p<0.001]. As predicted, phagocytosis of yeast was inhibited for neutrophils treated with the non-hydrolyzable cAMP analog 8-bromo-cAMP [1 µM ; ANOVA: p<0.001]. This method discriminates between FITC-stained HKO *C. albicans* engulfed by the neutrophils and those that are simply adherent to the outer neutrophil membrane. Representative images using light and epi-fluorescence microscopy of neutrophils engulfing the yeast are shown in Figure 3A. As a consequence of phagocytosing *Candida*, apoptosis is rapidly induced in the infiltrating neutrophils, [28], [29] thereby providing a potential mechanism through which the rate of neutrophil elimination from the site of infection may increase.

We also tested whether the RvE1-dependent increase in neutrophil phagocytosis of *Candida* is correlated with increased fungicidal activity. Neutrophils exposed to RvE1 exhibited a positive dose-dependent increase in their ability to kill opsonized *C. albicans* (Figure 3C). Twice as many opsonized *C. albicans* were killed by neutrophils treated with 1nM RvE1 and over three times as many yeast were killed by neutrophils treated with 10nM RvE1 relative to vehicle treated cells [ANOVA: For 10nM RvE1, p<0.001]. Neutrophils generate intra- and extra-cellular ROS such as hydroxy-radicals and super-oxide to kill invading pathogens [30]. Neutrophils exposed to 100nM RvE1 and HKO *C. albicans* produced more hydroxy-radical relative to neutrophils exposed to 10 or 1nM RvE1 (Figure 3D: orange or yellow circle, respectively) or vehicle-treated cells (Figure 3D: blue diamond). But RvE1 had no effect on neutrophil super-oxide production relative to the vehicle-treated neutrophils exposed to HKO *C. albicans* (Figure 3E). In the absence of HKO *C. albicans*, 100 nM RvE1 did not increase the amount of hydroxy-radical or super-oxide produced by neutrophils, compared to vehicle-treated cells (Figure 3DE: black circle). Neither vehicle, nor lower concentrations of RvE1 increased neutrophil ROS production in two of three neutrophil isolates (Figure 3DE and Figure S5ABC). However, for a single neutrophil isolate (Figure S5D), super-oxide production was elevated in neutrophils exposed to *Candida* and 1 or 10 nM RvE1, suggesting that there may be donor-dependent variations in neutrophil responses to RvE1.

### RvE1 reduces *C. albicans* levels *in vivo*

**Figure 4:**
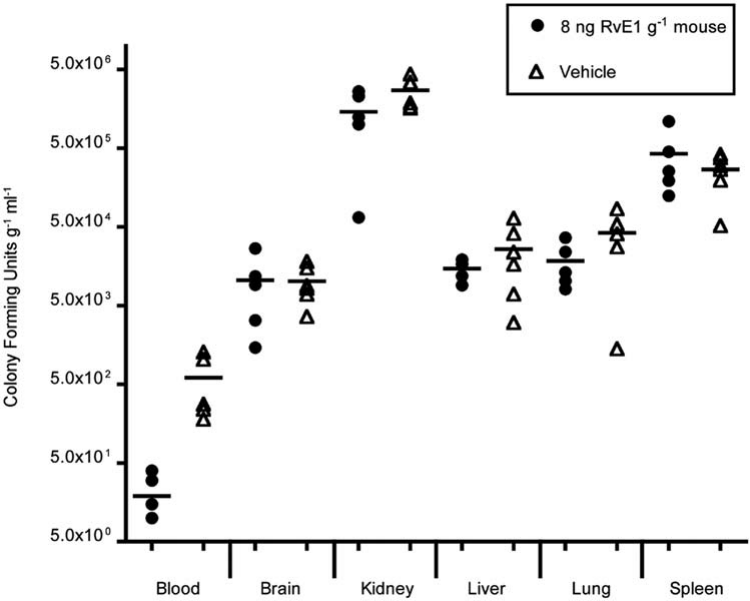
RvE1 reduces *C. albicans* concentrations in the blood. To evaluate the actions of RvE1 on the virulence of *C albicans*, 8–10 week old female BALB/c mice were injected via the tail vein with RvE1 (8 ng g^−1^ mouse) or vehicle and *C. albicans* (5×10^4^ yeast cells g^−1^ mouse). After 24 h, and blood and organs were collected, homogenized, serially diluted and plated. There was a 10-fold reduction in *C. albicans* circulating in the blood of mice injected with RvE1 compared to those injected with vehicle (circles vs triangles). In contrast, RvE1 and vehicle-treated mice showed similar levels of organ colonization.

To evaluate the actions of RvE1 on hematogenous infections of *C. albicans*, 8–10 week old female BALB/c mice were injected via the tail vein with RvE1 (8 ng g^−1^ mouse) or vehicle and *C. albicans* (5×10^4^ yeast cells g^−1^ mouse). Animals were sacrificed after 24 h, and blood and organs were collected. *Candida* colony forming units were enumerated as described in the Methods. At the *Candida* dosage used, the 24 h time point corresponds with the beginning of exponential growth of the fungus in the brain and kidneys of BALB/c mice while remaining detectable in other organs and blood [31]. We observed a 10-fold reduction in *C. albicans* circulating in the blood of mice treated with RvE1 compared to those treated with vehicle (Figure 4; circles vs triangles; Mann-Whitney Test, p = 0.0079). In contrast, RvE1-treated and vehicle-treated mice showed similar levels of organ colonization (Mann-Whitney Test, p>0.1220 for all paired tests).

## Discussion

In this study, we have characterized the effects of RvE1 on neutrophils in association with one of their primary pathogen targets, *C. albicans*. RvE1 is one of several potentially immuno-regulatory lipids produced *de novo* by *C. albicans* cultured in the presence of the omega-3 fatty acids, EPA and DHA. From these fatty acid substrates, *C. albicans* biosynthesizes resolvins and protectins, which in humans inhibit neutrophil migration and protect tissues from leukocyte-mediated inflammation [2], [8], [32]. Enzymatically-modified lipids have deep evolutionary origins as signaling molecules and are possible progenitors of innate immune responses. In humans, biosynthesis of RvE1 occurs in a *trans*-cellular process [33]. Hypoxic human endothelial cells donate 18R-hydroxyeicosapentaenoic acid (18R-HEPE), which is oxygenated by neutrophil 5-lipoxygenase (LO) and in subsequent enzymatic steps, converted to RvE1 [14]. In contrast, *C. albicans* is able to biosynthesize nanogram quantities of this anti-inflammatory lipid *de novo* from EPA and without the collaboration of other cellular partners. Although candidate enzymes which perform this synthesis are not obvious from *in silico* analysis of *C. albicans,* its genome encodes at least fifteen CYP450s, suggesting that one or more biosynthetic steps in fungal RvE1 could occur via these enzymes. It is reported that 18*R-*HEPE of microbial origin in gastrointestinal tissues may serve as a substrate for the production of RvE1, thereby dampening the host immune response which would otherwise be damaging to both microbe and host [15]. Oxygenated precursors to resolvins and protectins were detected when *C. albicans* was incubated with EPA or DHA, indicating that *Candida* can also contribute to resolvin and protectin synthesis by providing oxygenated substrates to host cells.

Both *Candida* and RvE1 modulate innate immune system functions. Human epithelial and endothelial cells infected by *C. albicans* release IL-8, which serves to attract neutrophils to sites of inflammation [24], [26], [27]. Release of membrane lipids follows immune cell activation [34], and may provide substrates used by *Candida* for RvE1 biosynthesis; alternatively dietary sources of EPA and DHA may also serve as substrates for resolvin biosynthesis. Our studies suggest that at sites of commensal colonization, low quantities of RvE1 biosynthesized by *Candida* would inhibit IL-8-mediated neutrophil chemotaxis (Figure 2). RvE1 also reduces the migration of antigen-presenting dendritic cells (DC) and inhibits stimulated DC interleukin-12 synthesis which may down-regulate T-lymphocyte responses to the antigen-stimulated DC, thereby dampening the adaptive immune response against *Candida* [10]. Together these activities would serve to protect the resident yeast cells from clearance by the innate and adaptive immune system. Thus the synthesis of RvE1 by *Candida,* resident on the host mucosa in small numbers as a commensal organism, may functionally sequester the fungus from host innate surveillance.

Local production of RvE1 by *Candida* potentially deters the migration of neutrophils to sites of inflammation while enhancing the killing functions associated with neutrophils, including phagocytosis, ROS synthesis and fungicidal activity (Figure 2 and Figure 3, Figure S5 and Figure S6). In a murine model of systemic candidiasis, a high dosage of RvE1 reduced numbers of fungi circulating in the blood 24 h after infection. Interestingly, higher levels of RvE1 are not as effective at inhibiting neutrophil chemotaxis as are lower levels, suggesting that fungi in more heavily colonized tissue, such as that associated with the onset of invasive disease, would not benefit from dampening the host innate response, but rather would be increasingly susceptible to neutrophil killing. Thus the overall effect of RvE1 may be governed by the number of fungi present, their physical location in tissues and the timing of RvE1 biosynthesis (Figure S6). In this regard, recent data indicates that vaginal candidiasis is correlated with both the presence of large numbers of *Candida* and the activation of an overzealous granulocyte response [35] that is otherwise dampened during commensal carriage. Similarly, reduction of microbial flora by antibiotic therapy is a risk factor for *Candida*-mediated esophageal and gastrointestinal inflammation [36], suggesting that host tolerance is modulated by fungal load. In this scenario, as yeast cells increase in number and invasiveness, the protective effect of RvE1 would wane and the neutrophils recruited to the site of active infection would be more effective in controlling virulent growth. Neutrophils would be very effective in this regard, as these are the only cells in the innate immune system that are able to engulf and kill the more invasive hyphal forms of the fungus [22]. Thus, RvE1 produced by *Candida* would, on the one hand, protect the yeast forms of the fungus, while higher concentrations would be ineffective in protecting the hyphal forms.

*C. albicans* biosynthesizes not only RvE1, but also a number of other oxygenated products with known biological activity in humans (Table S1). Coupled with the phylogenetic conservation of biologically active oxygenated lipids in plants and animals, and the detection of eicosanoids such as prostaglandin E2 in pathogenic fungi [37], [38], our findings show that oxygenated lipids such as the resolvins and protectins are produced by a fungal pathogen. These lipid mediators function as components of a complex and untapped chemical signaling system that underlies a fundamentally new paradigm of interaction between host and pathogen.

## Materials and Methods

### Strains and media

For all studies, *C. albicans* strain SC5314, maintained at −80°C, was plated on agar plates containing YPD (10 g l^−1^ yeast extract, 20 g l^−1^ Bacto-peptone, 20 g l^−1^ dextrose) and subsequently grown at 30°C in liquid YPD media before each experiment.

### Isolation and LC-MS/MS analysis of oxygenated lipids from *C. albicans*

For LC-MS/MS analysis of oxygenated lipids, 10^7^ *C. albicans* yeast were inoculated into 25 ml of liquid YNB+CSM media (Bio101, Inc.; Carlsbad, CA) adjusted to pH 6.8 and supplemented with 2% dextrose or 0.2% (v/v) EPA (Fluka) plus 0.02% dextrose and grown at 30°C in a rotary shaker (225 RPM) for 72 h. After harvest, cell pellets were washed 3× with phosphate buffered saline (PBS; 0.1 g l^−1^ CaCl_2_, 0.1 g l^−1^ MgCl_2_ 6H_2_O, 0.2 g l^−1^ KCl, 0.2 g l^−1^ KH_2_PO_4_, 8.0 g l^−1^ NaCl, 2.16 g l^−1^ Na_2_HPO_4_ 7H_2_O). 3×10^8^ cells per sample were suspended in 3 ml of sterile-filtered liquid YNB media adjusted to pH 6.8 supplemented with 2% dextrose or 15 µg/ml EPA. After incubating for 30 m (37°C, 80 RPM) the cultures were centrifuged for 10 m (2500×g), supernatants and cell pellets separated, snap-frozen in liquid nitrogen and stored at −80°C. Oxygenated lipids were extracted and analyzed with LC-MS/MS as previously described [39].

### Neutrophil isolation

Peripheral venous blood was obtained by venipuncture from healthy volunteers who reported to have abstained from taking any medication for at least two weeks prior to venipuncture (in accordance with the University of California, San Francisco Committee on Human Research (Approval Number H2430-24592-02), patients were informed of the risks of the procedure and provided oral consent before venipuncture); blood was collected into heparinized tubes and centrifuged for 10 m (150×g) at room temperature. The lower layer of cells was subjected to dextran sedimentation (Dextran T500; Fisher Scientific) for 20 m to separate the red blood cells (RBC). The upper leukocyte-containing fraction was further enriched for neutrophils by centrifugation in a Histopaque 1077/1119 (Sigma-Aldrich) step gradient. After lysis of residual RBC (ACK lysis buffer; Invitrogen) and washing with PBS lacking calcium or magnesium, neutrophils were suspended in RPMI-1640 medium (Cell Culture Facility; UCSF) or PBS and used for experiments within 1 h. Prepared in this way, >95% of the cells in the final fraction were neutrophils. All statistical analyses were made using the statistical program, GraphPad InStat 3.0b.

### Neutrophil migration

The chemotactic activity of synthetic RvE1 and the ability of RvE1 to inhibit IL-8-mediated chemotaxis was evaluated by measuring neutrophil migration through 3.0 micron pores of polycarbonate membranes in 24-well Transwells (Corning Costar; Corning, NY). Neutrophils (10^6^) suspended in RPMI-1640 were added to the top chamber and various concentrations of RvE1, vehicle (<0.1% ethanol) and/or IL-8 were added to the bottom well. To assess fugetactic or chemokinetic activity, RvE1 was placed in the top or top and bottom chambers of the Transwell plate, respectively. When assessing the inhibitory activity of RvE1 on IL-8-mediated chemotaxis, RvE1 or vehicle was added to the neutrophils suspension 5 m prior to their addition to the upper chamber of the Transwell. After 45 m of incubation at 37°C with 5% CO_2_, EDTA (7mM final concentration; Sigma-Aldrich) was added to the lower chamber to release neutrophils adhering to the membrane and bottom of the well. Microscopy was used to confirm that neutrophils were not adherent and the cells in four aliquots from each sample was counted using a hemocytometer. Results were reported as chemotactic index (the number of cells migrating to the lower chamber in response to a test-substance divided by the number of cells migrating spontaneously to the lower chamber in response to the vehicle).

### Neutrophil phagocytosis of *Candida*

Neutrophil phagocytosis of *C. albicans* was assessed as previously described [40], with modifications. Briefly, yeast were heat killed, washed twice with PBS, counted and suspended in PBS (3×10^8^ cells ml^−1^). Yeast were subsequently opsonized with 25% human serum for 30 m at 37°C, washed three times with PBS, stained in PBS containing 1.5 µg/ml fluoroscein isothiocyanate (FITC; Sigma) for 30 m at 4°C, washed three times with PBS, suspended in PBS and frozen at −20°C until use. With this method, 98% of the *Candida* remained in the yeast form and did not form germ tubes. Isolated human neutrophils suspended in RPMI-1640 medium were mixed with the test compound or vehicle (<0.1% ethanol) and incubated for 5 m. Neutrophils were added to 22 mm coverslips (Corning) contained within a 6-well plate (10^5^ neutrophils per well) and immediately mixed with FITC-stained heat-killed opsonized (HKO) *C. albicans* (3×10^5^) and incubated for 1h (37°C, 5% CO_2_). After incubation, plates were placed on ice and to quench the fluorescence of non-phagocytosed yeast, an equal volume of ice-cold trypan blue (250 µg/ml in 0.1 M citrate buffer, pH 4.0; Fluka) was added to each well, incubated for 1m on ice and the cover slips were subsequently inverted and mounted onto slides for viewing with an inverted microscope (200×). Neutrophils within 10 randomly-selected fields of view were scored for the presence or absence of phagocytosed FITC-stained HKO *C. albicans* (n = 200–300 neutrophils counted per sample).

### ROS generated by neutrophils

Studies of ROS production were performed as previously described [30], with modifications. Briefly, adherent neutrophils were treated with RvE1 or vehicle (<0.1% ethanol) for 5 m in the presence of 100 µM lucinigen or 1 µM luminal (Sigma-Aldrich) and then synchronously exposed to HKO *C. albicans* (moi = 5) with centrifugation (800×g for 5 m). Cells were subsequently incubated at 37°C, and chemiluminescence was measured every 2 m for 60 m using a luminometer (TR 717 Microplate Luminometer, Applied Biosystems).

### Neutrophil killing of *Candida*

Fungicidal activity of neutrophils was evaluated as previously described [41], with modifications. *C. albicans* was grown in liquid YPD medium, as described above, washed twice with PBS, counted and 5×10^9^ cells/ml were opsonized with 25% human serum (30 m, 37°C). Opsonized yeast were washed three times with PBS, suspended in cold RPMI-1640 and stored on ice until use. With this method, 98% of the *Candida* remained in the yeast form and lacked germ tubes. Isolated human neutrophils were incubated on ice with the test compound for 5 m and subsequently mixed with opsonized *C. albicans* (25 neutrophils : 1 yeast) in a 6-well plate (Corning Costar). After incubation for 2 h at 37°C, plates were centrifuged (20 m, 2500×g), water (pH 11) added to lyse the neutrophils and using a cell scraper, adherent yeast were detached from the surface of the plate. After scraping, plates were examined with an inverted microscope to ensure that adherent yeast had been collected. To determine the number of surviving yeast, each sample was serially diluted in PBS, spread onto YPD agar plates, incubated for 36 h at 30°C and resulting colony forming units (CFU) counted. Each experiment was performed in triplicate and each dilution was plated in duplicate. *Candida* killing by neutrophils is shown as fold-increase of RvE1-treated neutrophils over vehicle-treated neutrophils.

### Virulence studies

The study was approved by the University of California, San Francisco Institutional Animal Care and Use Committee (UCSF IACUC) prior to study initiation (IACUC protocol #: A2430-07582). In addition, UCSF Biosafety Committee (BSC) approved the use of *C. albicans* in this animal model to induce systemic candidiasis (BSC protocol #: 4BU 08 BAC). 6-week-old female BALB/c mice were purchased from Charles River Laboratories, (Wilmington, MA) and housed under specific-pathogen-free conditions at the University of California, San Francisco Laboratory Animal Care Facility. Liquid cultures of *C. albicans* were washed, suspended in ice-cold PBS, and counted. Cell concentration was adjusted with sterile PBS. Microscopic examination showed the cell suspension to be predominantly composed of single cells, with minimal clumping. Immediately prior to injection, the yeast suspensions were mixed, warmed to 30°C and loaded into 30cc insulin syringes fitted with a 31gauge needle. Prior to inoculation, mice were weighed (average mass = 20.6 +/− 1.2 g) and warmed on heating pads. Mice were first inoculated via the right tail vein with 8ng of RvE1 per gram of mouse or vehicle (<0.1% ethanol) diluted in sterile PBS. Subsequently, the yeast suspension was introduced into the left tail vein, delivering a total of 5×10^4^ yeast cells per gram of mouse. 24 h after injection, mice were sacrificed and blood, brain, left kidney, liver, left lung, and spleen processed to determine fungal burden in these tissues. To determine the number of viable *C. albicans* cells, the collected blood volume was measured and organs were weighed, homogenized, diluted with PBS, and quantitatively cultured on YPD agar at 37°C for 2 days.

## Supporting Information

Figure S1 Growth characteristics of *C. albicans* cultured in glucose or complex oils. *C. albicans* was cultured in liquid YNB+CSM media supplemented with 2% glucose or 0.2% complex oil plus 0.2% glucose (30 °C, 225 RPM) and fungal growth estimated by measuring optical density (OD600). Similar growth characteristics were observed when estimated by measuring accumulation of dried fungal mass (not shown).
(1.50 MB TIF)

Figure S2 Inhibitors of LO and CYP450 do not inhibit *Candida* growth. *C. albicans* grew to similar cell densities when cultured in the presence of EPA +/−inhibitors (initial culture conditions: 2×10̂6 yeast cells were suspended in 100 ml of liquid media (YNB+CSM adjusted to pH 6.8 and supplemented with 2% dextrose or 0.2% (v/v) EPA plus 0.02% dextrose and with inhibitor (10 µM) or ethanol vehicle (>0.1%)) and cultured for 72h at 30 °C and 225 rpm. When cultured in dextrose, the fungus grew to higher densities in the presence of each inhibitor, although the differences between inhibitor and vehicle treated cultures were not statistically significant (ANOVA; p>0.05)
(1.24 MB TIF)

Figure S3 Inhibition of neutrophil IL-8 chemotaxis by RvE1. For neutrophils isolated from two additional donors (AB), IL-8-directed chemotaxis of neutrophils was significantly inhibited by 10 and 100 nM RvE1 (Asterisks indicate significant differences from vehicle-treated controls; ANOVA: p<0.001).
(2.31 MB TIF)

Figure S4 RvE1 is not chemotactic, fugetactic, or chemokinetic to human neutrophils. 100nM leukotriene B4 (LTB4) in the lower chamber of the transwell was a strong neutrophil attractant (chemotactic index (CI) = 30.3 +/− 5.3), three different concentrations of RvE1 (1 to 100 nM) in the lower chamber failed to induce directed neutrophil chemotaxis (CI<1.5), suggesting that RvE1 is not a neutrophil chemoattractant (ANOVA; p>0.05). To assess fugetactic activity, RvE1 was placed in the upper chamber with the neutrophils; there was no directed migration of neutrophils into the lower chamber suggesting that RvE1 does not repel neutrophils (CI<1.6; ANOVA; p>0.05). When RvE1 was placed in both the upper and lower chambers of the transwell, there was no directed migration of neutrophils indicating that RvE1 is not chemokinetic to neutrophils (not shown).
(0.18 MB TIF)

Figure S5 RvE1 effect on hydroxy-radical and superoxide produced by neutrophils exposed to *C. albicans*. (AB) For neutrophils isolated from two additional donors, RvE1 had no effect on hydroxy-radical produced by neutrophils exposed to HKO *C. albicans* (red, orange, and yellow circles vs blue diamonds). (CD) As with the first neutrophil donor (Figure 3DE), 100 nM RvE1 increased neutrophil superoxide production in neutrophils exposed to HKO *C. albicans* relative to neutrophils exposed to vehicle and HKO *C. albicans* (red circles vs blue diamonds). While for a second neutrophil isolate, lower concentrations of RvE1 (orange and yellow circles) did not increase neutrophil superoxide relative to cells exposed to vehicle and HKO *C. albicans* (blue diamonds), the third neutrophil isolate (Supplementary Figure 5D) displayed increased superoxide production for cells treated with 1 and 10 nm RVE1. For neutrophils isolated from all donors, 100nM RvE1 alone did not increase the amount of ROS produced by neutrophils relative to vehicle controls (black circles).
(1.12 MB TIF)

Figure S6 Model of potential local and distal actions of RvE1 generated by *C. albicans*. *C. albicans* colonizing human epithelial cell surfaces can metabolize host cell and dietary EPA using as yet unidentified fungal oxygenases. Fungal RvE1 may inhibit distal IL-8 mediated recruitment of neutrophils into the site of colonization. To control overgrowth of the fungus, local RvE1 enhances neutrophil phagocytosis and fungal killing.
(2.04 MB TIF)

Table S1 Oxygenated lipids produced by *C. albicans* cultured in omega-3 PUFA.
(0.20 MB DOC)
