# Supplementary figures and images for: Candida albicans Modulates Host Defense by Biosynthesizing the Pro-Resolving Mediator Resolvin E1
Source: PLoS One. 2007 Dec 19;2(12):e1316. doi: 10.1371/journal.pone.0001316 (PMC2134765; doi:10.1371/journal.pone.0001316)

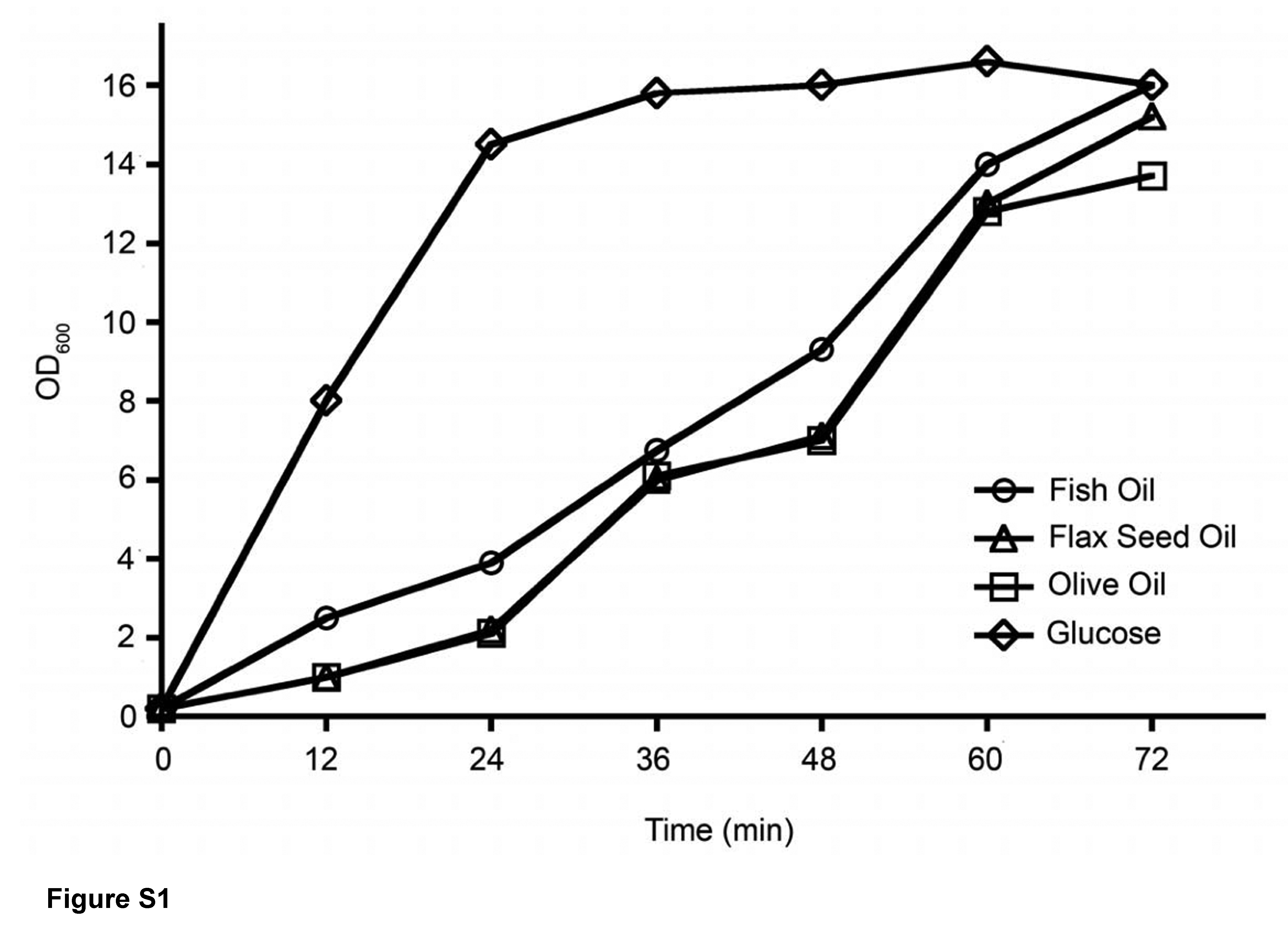

Supplement: Figure S1 — Growth characteristics of C. albicans cultured in glucose or complex oils. C. albicans was cultured in liquid YNB+CSM media supplemented with 2% glucose or 0.2% complex oil plus 0.2% glucose (30 °C, 225 RPM) and fungal growth estimated by measuring optical density (OD600). Similar growth characteristics were observed when estimated by measuring accumulation of dried fungal mass (not shown). (1.50 MB TIF) [file pone.0001316.s001.tif]

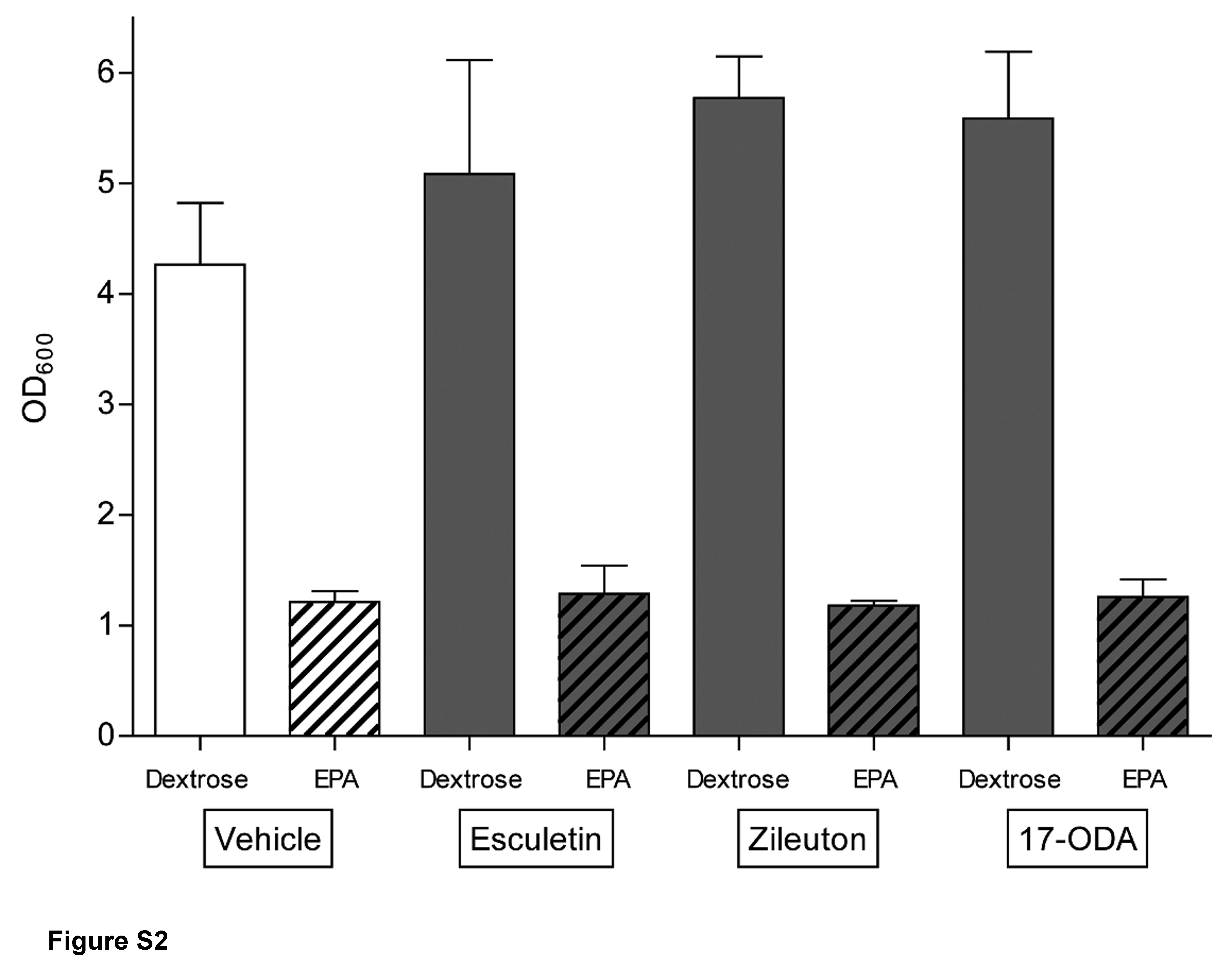

Supplement: Figure S2 — Inhibitors of LO and CYP450 do not inhibit Candida growth. C. albicans grew to similar cell densities when cultured in the presence of EPA +/−inhibitors (initial culture conditions: 2×10̂6 yeast cells were suspended in 100 ml of liquid media (YNB+CSM adjusted to pH 6.8 and supplemented with 2% dextrose or 0.2% (v/v) EPA plus 0.02% dextrose and with inhibitor (10 µM) or ethanol vehicle (>0.1%)) and cultured for 72h at 30 °C and 225 rpm. When cultured in dextrose, the fungus grew to higher densities in the presence of each inhibitor, although the differences between inhibitor and vehicle treated cultures were not statistically significant (ANOVA; p>0.05) (1.24 MB TIF) [file pone.0001316.s002.tif]

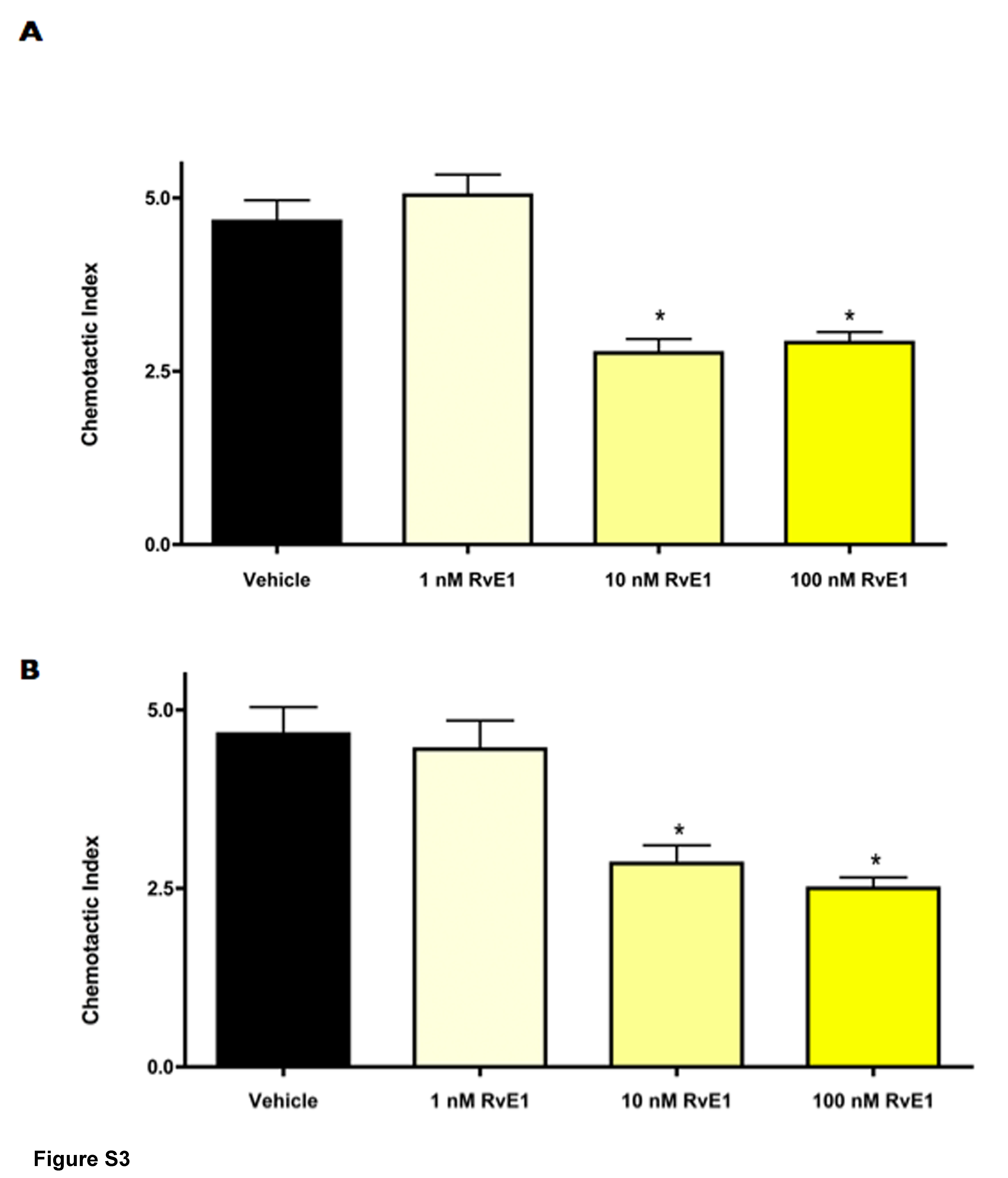

Supplement: Figure S3 — Inhibition of neutrophil IL-8 chemotaxis by RvE1. For neutrophils isolated from two additional donors (AB), IL-8-directed chemotaxis of neutrophils was significantly inhibited by 10 and 100 nM RvE1 (Asterisks indicate significant differences from vehicle-treated controls; ANOVA: p<0.001). (2.31 MB TIF) [file pone.0001316.s003.tif]

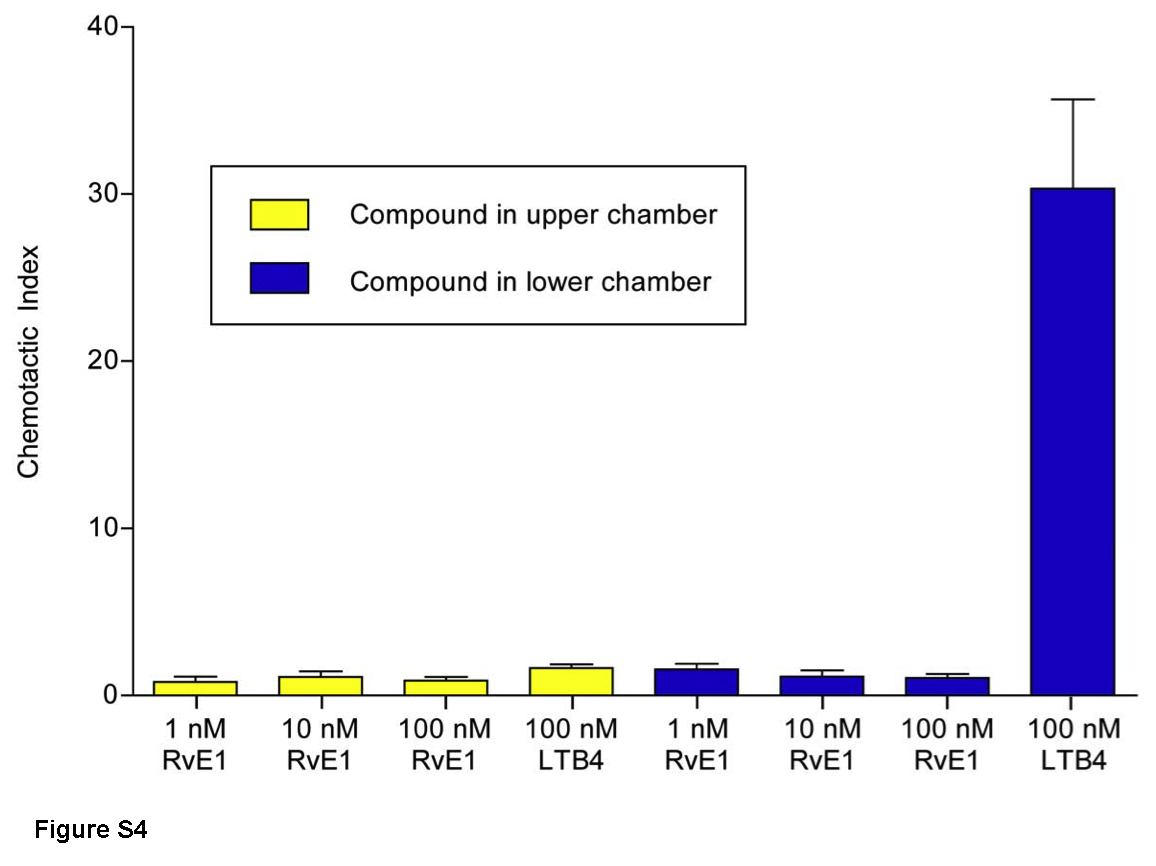

Supplement: Figure S4 — RvE1 is not chemotactic, fugetactic, or chemokinetic to human neutrophils. 100nM leukotriene B4 (LTB4) in the lower chamber of the transwell was a strong neutrophil attractant (chemotactic index (CI) = 30.3 +/− 5.3), three different concentrations of RvE1 (1 to 100 nM) in the lower chamber failed to induce directed neutrophil chemotaxis (CI<1.5), suggesting that RvE1 is not a neutrophil chemoattractant (ANOVA; p>0.05). To assess fugetactic activity, RvE1 was placed in the upper chamber with the neutrophils; there was no directed migration of neutrophils into the lower chamber suggesting that RvE1 does not repel neutrophils (CI<1.6; ANOVA; p>0.05). When RvE1 was placed in both the upper and lower chambers of the transwell, there was no directed migration of neutrophils indicating that RvE1 is not chemokinetic to neutrophils (not shown). (0.18 MB TIF) [file pone.0001316.s004.tif]

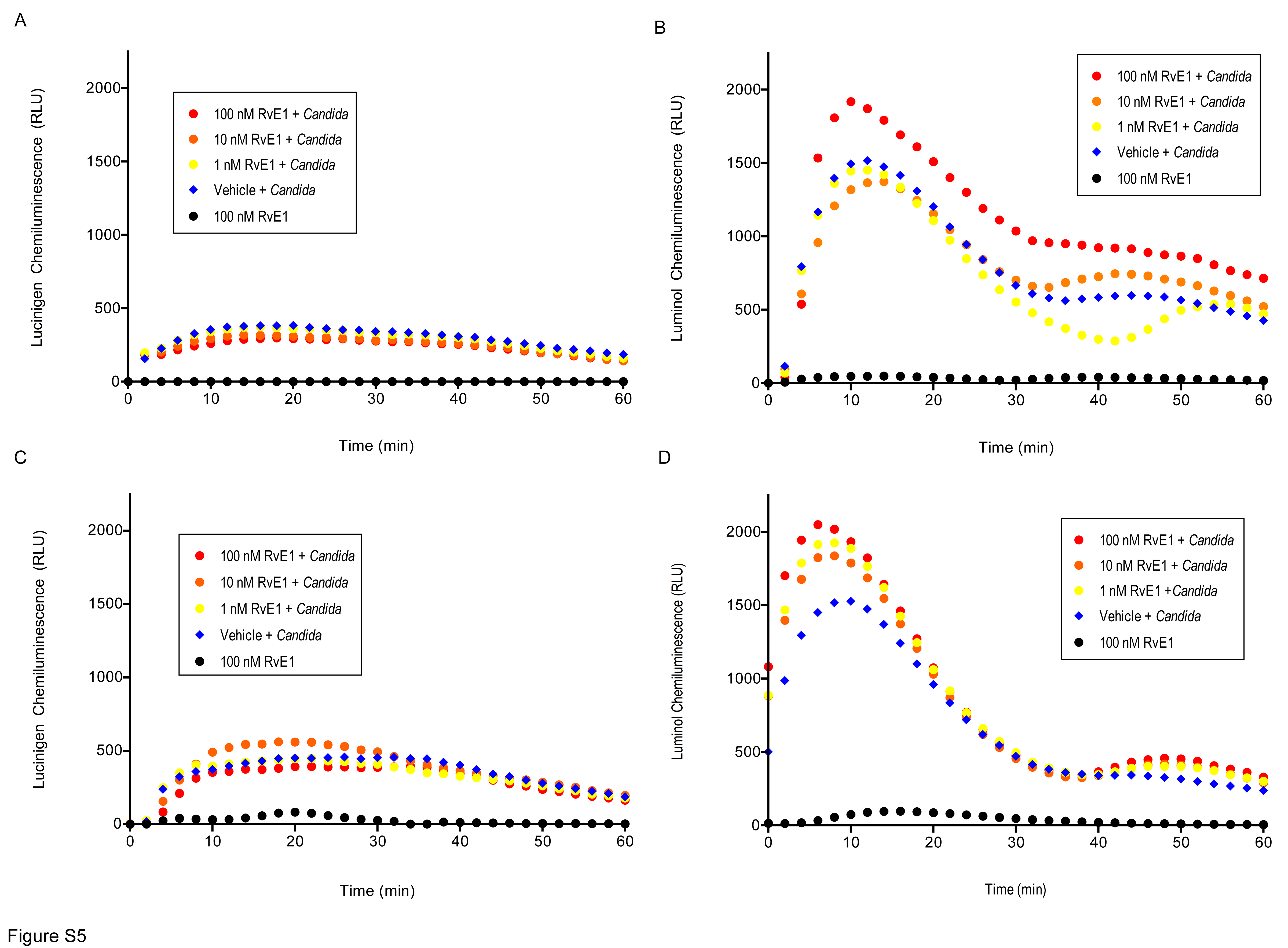

Supplement: Figure S5 — RvE1 effect on hydroxy-radical and superoxide produced by neutrophils exposed to C. albicans. (AB) For neutrophils isolated from two additional donors, RvE1 had no effect on hydroxy-radical produced by neutrophils exposed to HKO C. albicans (red, orange, and yellow circles vs blue diamonds). (CD) As with the first neutrophil donor (Figure 3DE), 100 nM RvE1 increased neutrophil superoxide production in neutrophils exposed to HKO C. albicans relative to neutrophils exposed to vehicle and HKO C. albicans (red circles vs blue diamonds). While for a second neutrophil isolate, lower concentrations of RvE1 (orange and yellow circles) did not increase neutrophil superoxide relative to cells exposed to vehicle and HKO C. albicans (blue diamonds), the third neutrophil isolate (Supplementary Figure 5D) displayed increased superoxide production for cells treated with 1 and 10 nm RVE1. For neutrophils isolated from all donors, 100nM RvE1 alone did not increase the amount of ROS produced by neutrophils relative to vehicle controls (black circles). (1.12 MB TIF) [file pone.0001316.s005.tif]

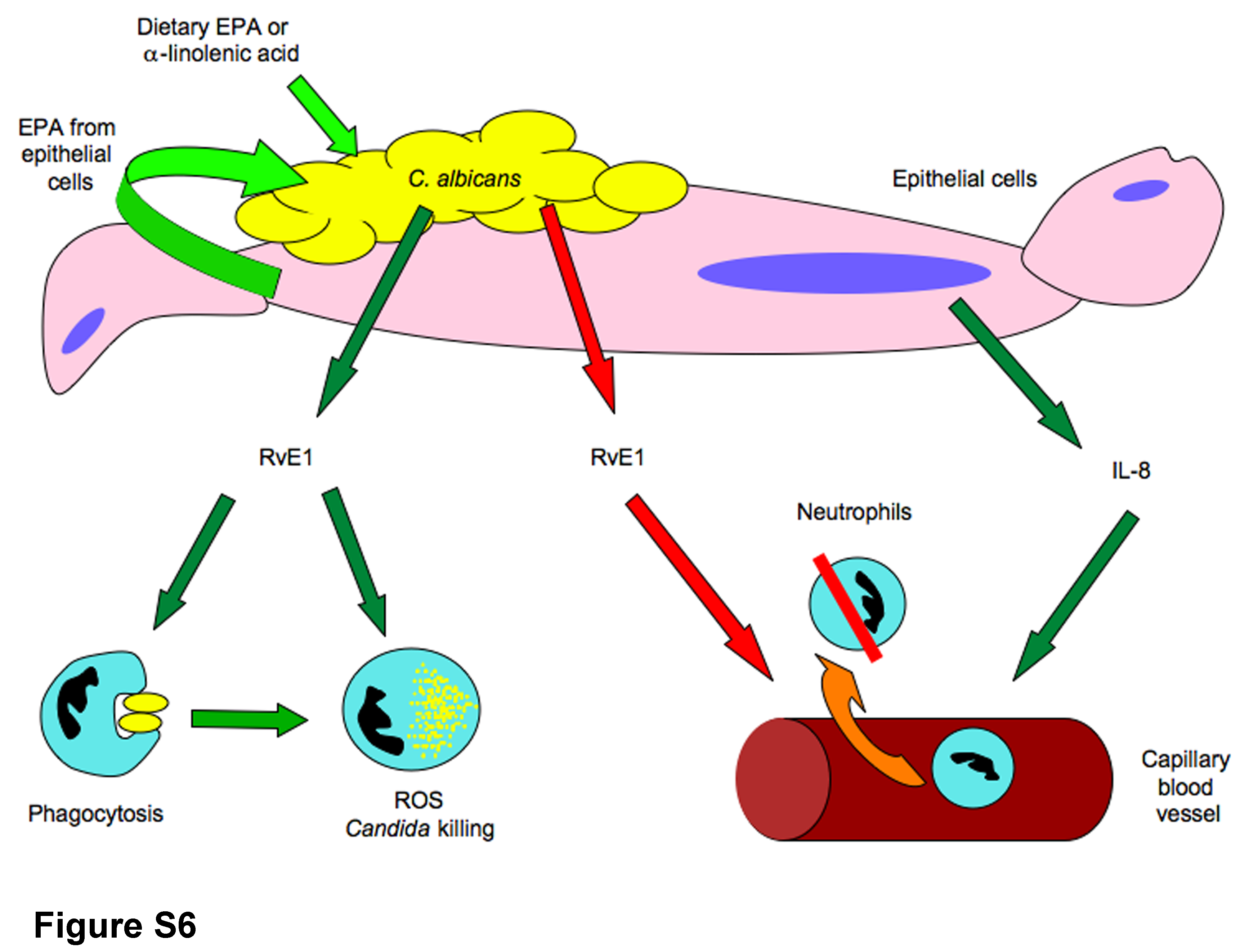

Supplement: Figure S6 — Model of potential local and distal actions of RvE1 generated by C. albicans. C. albicans colonizing human epithelial cell surfaces can metabolize host cell and dietary EPA using as yet unidentified fungal oxygenases. Fungal RvE1 may inhibit distal IL-8 mediated recruitment of neutrophils into the site of colonization. To control overgrowth of the fungus, local RvE1 enhances neutrophil phagocytosis and fungal killing. (2.04 MB TIF) [file pone.0001316.s006.tif]
