# Supplementary material for: Candida albicans Modulates Host Defense by Biosynthesizing the Pro-Resolving Mediator Resolvin E1
Source: PLoS One. 2007 Dec 19;2(12):e1316. doi: 10.1371/journal.pone.0001316 (PMC2134765; doi:10.1371/journal.pone.0001316)
Supplement: Table S1 — Oxygenated lipids produced by C. albicans cultured in omega-3 PUFA. (0.20 MB DOC) [file pone.0001316.s007.doc]

|  | | | |
| --- | --- | --- | --- |
|  | | | |
|  |  |  |  |
| DHA derived products in cultures with DHA or code fish oil | RT (min) | Ions | Structures |
| RvD3 | 6.8 | 375, 357, 339, 273 | **HO**  **OH**  **OH**  **COOH** |
| RvD4 | 7.2 | 375, 357, 339, 233 | **HOOC**  **OH**  **OH**  **OH** |
| RvD2 | 4.8 | 375, 357, 339, 247 | **COOH**  **OH**  **OH**  **HO** |
| NPD1 | 11.4 | 359, 341, 315, 153, 181 | OH  OH  COOH |
| 17-hydroxy DHA | 21.0 | 343, 325, 245 |  |
|  |  |  |  |
| EPA derived products in derived products in cultures with EPA or code fish oil |  |  |  |
| RvE1 | 5.1 | 349, 331, 195, 291 | OH  COOH  HO  OH |
| 18-HEPE | 16.3 | 317,259 |  |
| 15-HEPE | 16 | 317,219 |  |
| 11-HEPE | 18 | 317,195 |  |
| 12-HEPE | 18 | 317,179 |  |
| 9-HEPE | 20 | 317,167 |  |
| 5-HEPE | 22.4 | 317,115 |  |

**Table S1**
